# Emergency Department presentations with suicidal ideation: A missed opportunity for intervention?

**DOI:** 10.23889/ijpds.v8i2.2230

**Published:** 2023-09-14

**Authors:** Emma Ross, Aideen Maguire, Denise O'Hagan, Dermot O'Reilly

**Affiliations:** 1 Queen's University Belfast, Belfast, United Kingdom; 2 Public Health Agency Northern Ireland, Belfast, United Kingdom

## Objectives

Suicidal ideation constitutes a central element of most theories of suicide. Despite its prevalence, most research has focused on other suicidal behaviours such as self-harm. This study examines the characteristics of those presenting to EDs with suicidal ideation and quantifies the associated risk of suicide and other causes of death.

## Method

This retrospective cohort study used population-wide health administration data linked to data from the Northern Ireland Registry of Self-Harm and centrally held mortality records from April 2012 to December 2019. Mortality data, coded as suicide, all-external causes and all-cause mortality was analysed using Cox proportional hazards. Additional cause-specific analyses included accidental deaths, deaths from natural causes, and drug and alcohol-related causes.

## Results

The final cohort comprised 1,662,118 individuals aged over 10 years, of whom 15,267 presented to the ED with ideation during the study period. Individuals with ideation had a ten-fold increased risk of death from suicide (HRadj=10.84, 95% CI 9.18, 12.80) and from all external causes (HRadj=10.65, 95% CI 9.66, 11.74) and a three-fold risk of death from all causes (HRadj= 3.01, 95% CI 2.84, 3.20). Further cause-specific analyses indicated that risk of accidental death (HRadj=8.24, 95% CI 6.29, 10.81), alcohol-related death (HRadj=10.57, 95% CI 9.07, 12.31), and drug-related death (HRadj=15.17, 95% CI 11.36, 20.26) were also significantly raised. There were few socio-demographic and economic characteristics that would identify which of these patients are most at risk of suicide or other causes of death.

## Conclusion

The identification of those experiencing ideation is recognised to be important yet difficult in practice. The ED represents an important potential intervention point for this hard-to-reach population. However, and unlike those presenting with self-harm, clinical guidelines for the management and recommended best practice and care of these individuals are lacking.

